# Tooth Loss and Associated Factors in Mexican Older Adults in Nursing Homes: A Multicenter Cross-Sectional Study

**DOI:** 10.1155/2023/4169097

**Published:** 2023-04-15

**Authors:** Jesús Alberto Rocha-Ortiz, Sandra Manuela Tepox-Puga, S. Aída Borges-Yañez, Martha Mendoza-Rodríguez, Mauricio Escoffié-Ramirez, Mirna Minaya-Sánchez, Juan Fernando Casanova-Rosado, Alejandro José Casanova-Rosado, América Patrícia Pontigo-Loyola, Carlo Eduardo Medina-Solis

**Affiliations:** ^1^Master and Doctoral Program in Medical, Dental and Health Sciences at the School of Dentistry, National Autonomous University of México, 04510 Mexico City, Mexico; ^2^Dental Public Health Department, Graduate and Research Division at the School of Dentistry, National Autonomous University of México, 04510 Mexico City, Mexico; ^3^Academic Area of Dentistry of Institute of Health Sciences at Autonomous University of Hidalgo State, 42160 Pachuca, Mexico; ^4^School of Dentistry, Autonomous University of Yucatán, 97000 Mérida, Mexico; ^5^School of Dentistry, Autonomous University of Campeche, Campeche 24039, Mexico; ^6^Advanced Studies and Research Center in Dentistry “Dr. Keisaburo Miyata” of School of Dentistry at Autonomous University State of Mexico, Toluca, Mexico

## Abstract

The objective of this study was to determine the experience of tooth loss and associated factors in older adults and elderly residing in nursing homes. A cross-sectional study was conducted in Mexican older adults and elderly aged ≥60 years living in four nursing homes (two in Mexico City, Mexico: one in Cuernavaca, Morelos, and one in Oaxaca, Oaxaca). The data were collected at the facility (home nursing) by two dentists in 2019. To determine the number of tooth loss and DMFT, a clinical oral examination was performed. In addition, a questionnaire was applied to determine diverse independent variables (demographic, socioeconomic, and behavioral). The analysis was performed using nonparametric tests and negative binomial regression (*p* < 0.05). 257 subjects were included. The mean age was 81.25 ± 9.02 years, and 60.7% were women. The mean number of lost teeth was 18.78 ± 9.05 (women = 19.43 ± 8.59 and men = 17.77 ± 9.68; *p* > 0.05). In the multivariate negative binomial regression model, it was found that, for each one-year increase in age, the mean tooth loss increased 0.92% (*p* < 0.05). In current smokers (*p* < 0.01) and in those who brush their teeth < 2 times a day (*p* < 0.01), the average of tooth loss increased 22.04% and 61.46%, respectively. The experience of tooth loss in Mexican older adults and elderly was high. Demographic (age) and habit of behavior (tobacco use and less frequent tooth brushing) were associated with increased tooth loss. It is important to promote oral health programs for institutionalized older adults.

## 1. Introduction

The proportion of older adults continues to rise worldwide [1]. In Mexico, one of the most pressing challenges is the aging of the population; this situation is associated with a high risk of death, disability, poor quality of life, loss of functionality, and institutionalization, which translates into a greater number of institutionalized older adults [2, 3]. In addition, oral health is also impaired. Among institutionalized older adults, there is a high prevalence of caries, periodontal disease, tooth loss, need for dental prosthesis, oral mucosal lesions, poor oral hygiene, and dental calculus, among others [4]. Despite nursing homes must provide routine dental care, the oral care received by institutionalized older adults is inadequate [5, 6]. The absence of dental care increases the risk of medical complications. For example, tooth loss leads to changes in food choice, which in turn triggers nutritional problems and affects the general health of older adults [7, 8].

Tooth loss is a complex result that reflects the history of a person's dental disease and its treatment, in addition to the attitude of the patient and the dentist towards the disease, the dentist-patient relationship, the availability and accessibility to dental services, and even the treatment philosophy in force at the time [9]. Due to the negative impact it has on the quality of life, the large number of people affected and the cost necessary for their care, it is considered a public health problem. [1, 10]. According to the “Global Burden of Disease Study,” edentulism (complete loss of natural teeth) is an important problem for health of population. In 2017, the global age-standardized prevalence of edentulism was 3.3%, and there were 267 million of edentulous people worldwide. The more economically developed countries have the highest burden of tooth loss; however, it is relevant to note that the prevalence of edentulism has decreased in more economically developed countries and has increased in less economically developed countries from 1990 to 2017 [11]. According to a national survey in Mexico, approximately 10% of adults noninstitutionalized between 45 and 54 years old, 25% between 65 and 74 years old, and 30% between 65 and older are edentulous [12]. In a recent study, overall edentulism prevalence observed was 8.4% [13]. Regarding tooth loss, previous studies have observed the following results (mean age = 41.6 years): 52.7% had at least 1 tooth loss, with a mean of 2.9 tooth loss [14]. In another study (mean age = 42.45 years), the mean number of tooth loss was 7.46 [15]. On the other hand, in institutionalized subjects (mean age = 79.06), the mean number of tooth loss was 20.02 and 99.3% of the participants had at least 1 tooth loss [16]. Documenting trends in tooth loss may help in planning dental care services and workforce needs [7].

Inadequate oral hygiene favors the development of caries and periodontal disease, which are the main causes of tooth loss [4, 9]. Other causes of tooth loss are failure of endodontic treatment, trauma, dental fracture, and prosthetic or orthodontic reasons. Furthermore, extraction performed by the dental care provider is a common form of tooth loss, mainly in developing countries like Mexico [17, 18]. Epidemiological studies that have been carried out around the world have observed a series of factors related to the risk of tooth loss, for example, old age, being a woman, low educational level, low income, chronic diseases (diabetes and hypertension), oral diseases (caries and periodontal disease), presence of dental plaque and dental calculus, not having dental insurance, not visiting the dentist for prevention purposes, visit the dentist for dental pain, receiving radiation therapy, previous loss of teeth, tooth brushing less than twice a day, consuming sweet beverages, smoking, and taking certain medications [4, 18–21]. Little is known in Mexico about the extent of tooth loss in older adults and the factors that are associated. Therefore, the objective of this study was to determine the experience of tooth loss and associated factors in older adults and elderly residing in nursing homes.

## 2. Materials and Methods

### 2.1. Design and Study Population

A cross-sectional study was conducted in a group of older adults 60 years old and over residing in four nursing homes of the National System for the Integral Development of the Family: two in Mexico City, Mexico; one in Cuernavaca, Morelos; and one in Oaxaca, Oaxaca. The inclusion criteria were as follows: (1) any sex, (2) 60 years and older, (3) to be institutionalized in one of the 4 research sites, and (4) voluntarily participate and sign the informed consent. The exclusion criteria were as follows: (1) adults with any disease that prevented them from answering the questionnaire or performing the clinical oral exam and (2) older adults who presented severe cognitive impairment (serious behavioral problems, mobility, and communication problems).

All residents with no evidence of severe cognitive impairment were invited to participate. The purpose of the study was explained to them, and that participation was voluntary. Once the older adult and elderly reported no doubts about the project, they were given informed consent to be signed. We used the following parameters [16] to determine sample size: level of confidence (1 − *α*) = 95%, precision (*d*) = 1%, variance (*S*2) = 64, and 5% estimated losses, with a final sample of 259 subjects. A convenience sampling method was used, in which participation was voluntary.

### 2.2. Variables and Data Collection

Older adults and elderly institutionalized within gerontological centers were examined with the help of a headlamp, a reclining chair, a #5 mirror, a WHO-type periodontal probe, and a mobile dental unit. Data collection took place between July and December 2019. The dependent variable was the number of lost teeth and was determined by means of an oral clinical examination, using gloves, face masks, and a dental mirror. The root remains were considered as tooth loss. Third molars were excluded for this study. Clinical examinations were performed by qualified and trained dental surgeons. The pilot test was carried out in a group of 20 older adults who attended the “Day House for Older Adults” located in the Santa Ana Tlapaltitlán, Toluca, State of Mexico. To determine the degree of intraexaminer agreement and the degree of agreement between the standardized examiner and the examiner, the kappa test was used. The examiner agreement, obtained by calculating the kappa value for plaque and dental calculus, was 0.88, 0.78 for coronal caries, 0.89 for root caries, and 1.0 for evaluation of the functionality of dental prostheses.

A questionnaire was applied by an interviewer (master's student) face to face, to determine diverse demographic, socioeconomic, and behavioral variables, which were collected by a capacitated examiner, trained by an expert in the application of surveys with extensive experience in epidemiological studies. The independent variables included were as follows: demographic (age (years), gender (male/female), and marital status (without partner/with partner)); socioeconomic (schooling (more than secondary/secondary and less)); gerontological center of residence (Arturo Mundet (AM)/Vicente García Torres (VGT)/Los Tamayo (LT)/Olga Tamayo (OT) and health insurance (no/yes)); and behavioral variables (frequency of tooth brushing (2 or more times a day/<2 times a day) and number of sweets consumed in a day); current medical treatment (no/yes); a person was considered a smoker if they answered positively to the following question: do you smoke?; a person was considered as former smoker if they answered positively to the following question: have you been a smoker in past?; snacking between meals (no/yes); dental care in the last year (no/yes); and severe health problems that prevent following hygiene and care instructions (no/yes).

### 2.3. Statistical Analysis

In the univariate analysis, simple and relative frequencies were reported for the qualitative variables and measures of central tendency and dispersion for the quantitative variables. To search for the differences in the distribution of the number of tooth loss and the independent variables, a bivariate analysis was performed using nonparametric statistical tests (Spearman correlation, Mann-Whitney, and Kruskal-Wallis). A value of *p* < 0.05 was considered statistically significant.

Because the data does not fit the distribution for a Poisson regression model, that is, the variance was greater than the mean, we chose negative binomial regression (NBR) to adjust the multivariate model for tooth loss. Those variables with a *p* value < 0.25 in the bivariate analysis were included in the model to adequately control the effect of confounding factors. Through an analysis of the variance inflation factor (VIF), it was determined that there was no multicollinearity among the independent variables. Interactions were tested; however, none were found to be significant (*p* < 0.15) [22]. Confidence intervals with robust standard errors were calculated; since the data was obtained from older adults residing in different nursing homes (cluster), the observations within the groups could be correlated, while the observations among the groups were not [23]. Statistical analysis was carried out with Stata (StataCorp. 2017. Stata Statistical Sofware: Release 15. College Station, TX: StataCorp LLC).

### 2.4. Ethical Considerations

This study complied with the specifications of the General Health Law in Research in force in Mexico. All individuals signed an informed consent form. The protocol was approved at the School of Dentistry of the Autonomous National University of Mexico (CIE/0810/03/2017). Participants were informed that they could withdraw at any time and that no payment would be made for their participation in the study.

## 3. Results

The study sample consisted of 257 older adults. The mean age was 81.25 ± 9.02 years, and 60.7% were women. The descriptive analysis is shown in Table 1. Mean number of tooth loss was 18.78 ± 9.05 (median, 21). Just over one-third of the participants were edentulous (36.6%).

In the bivariate analysis (Table 1), it was observed that the mean number of tooth loss among women was 19.43 ± 8.59 and in men 17.77 ± 9.68 (*p* > 0.05). At this level of analysis, as age increased, the tooth loss also increased (positive correlation, *r* = 0.28; *p* < 0.001). In addition, no association was observed between the number of lost teeth and the number of sweets consumed in a day (*r* = −0.03; *p* = 0.621). Older adults with less schooling (*p* = 0.034), those who currently smoke (*p* = 0.007), and those who brush their teeth less than twice a day (*p* < 0.01) had a greater number of tooth loss.

Table 2 shows the results of the crude and adjusted negative binomial regression models. The crude negative binomial regression model for number of tooth loss showed an association with age, schooling, smoking, and frequency of tooth brushing. The final adjusted model showed that for each additional year of age, the expected mean number of tooth loss increased by 0.83% (*p* = 0.039). In older adults and elderly with less schooling, the expected average of tooth loss increased 10.65%, compared to those who have more schooling (*p* = 0.044). Furthermore, the expected mean number of tooth loss increased by 27.78% in smokers compared to nonsmokers (*p* = 0.004). Finally, in people who brushed their teeth less than twice a day, the expected average of tooth loss increased 65.13%, compared to those who brushed their teeth two or more times a day (*p* < 0.001).

## 4. Discussion

In this sample of institutionalized older adults, the average number of tooth loss was 18.78 ± 9.05, and demographic, socioeconomic, and health behaviors were associated with this event. The mean number of lost teeth was similar to that reported in Mexico [16] in institutionalized elder adults (mean number of tooth loss of 20.02), although higher than reported in other studies in younger noninstitutionalized subjects (mean of 2.9 tooth loss [14] and mean number of tooth loss of 7.46 [15]). However, these results differ from the results found in Brazil [24] where an average of tooth loss of 27.88 ± 6.8 was reported in institutionalized older adults, which is higher than the figure observed in this study.

One of the most consistent observations across epidemiological studies is that increased age is associated with tooth loss [16, 21, 24], as observed in this study. Although tooth loss is not an inherent result of aging, the explanation for why tooth loss is associated with age can be attributed to the accumulation of oral disease over the life span, mainly caries and periodontal disease [10, 16], progressive diseases, which lead to tooth loss if not treated [9]. On the other hand, it could be a reflection of the treatments that patients have received throughout their lives, that is, of the oral health services offered in Mexico, which are mainly interventionist and invasive [7, 11]. Likewise, we must consider that the participants of this study lived in a time in which accessibility to dental services was limited and the treatment philosophy was to extract teeth to prevent systemic diseases. Therefore, people may think that tooth loss is an inevitable fact of the aging process, especially those with little or no information on oral health [16, 25].

Concerning schooling, a lower level of education was associated with higher tooth loss, as reported in several studies [20, 26]. The literature suggests that the association between education and oral health could be mediated by oral health-related behaviors [27]. It is common to observe negative behaviors for oral health among people with lower educational level (tobacco use, high sugar consumption, not visiting the dentist regularly, and inadequate oral hygiene practices), which increases the risk of tooth loss [27–29]. Contrarily, it is likely that people with higher educational level to have positive attitudes and behaviors oral health-related behaviors because they understand the importance of oral care [4, 27]. Further, people with better education may have more information about oral health and prevention of oral diseases, which can avoid tooth loss [4].

In this study, tobacco use was associated with higher tooth loss; however, no association was found between the number of tooth loss and being former smoker. Similarly, Saito et al. [30] reported that smoking was associated with tooth loss in Japanese patients 65 years old or older, but there no was association between tooth loss and being former smoker. This result can be attributed to the positive effects of cessation smoking. Cessation smoking reduces the risk of developing periodontal disease, contributes to have better clinical outcomes after periodontal therapy, and reduces the risk of tooth loss. Moreover, after cessation smoking, the likely to lose teeth is similar among ex-smokers and people who have never smoked [31, 32].

Instead, there is solid evidence that demonstrates an association between smoking and tooth loss [31]. Tobacco use increases the risk of developing periodontal disease and contributes to its progression; this disease causes the destruction of the supporting tissues of the teeth, which initially results in clinical attachment loss, followed by the formation of pockets and bone loss, and concludes with tooth loss [10, 33]. Smokers are at higher risk of lose their teeth, as a result of a greater extent and severity of periodontal disease [32]. There is evidence that suggests that smoking promotes the presence of periodontal pathogens, as *Porphyromonas gingivalis*, in the subgingival microflora and affects host immunity, thus accelerating the destruction of the supporting tissues of the teeth. Some compounds in tobacco, such as nicotine, have adverse effects on the function of the cells responsible for the inflammatory and immune response. Besides, the microbial profile of people who smoke is characterized by a greater number of microorganisms that favor the appearance and progression of periodontal disease [33].

Furthermore, a lower frequency of tooth brushing was associated with higher tooth loss in this population. This association has previously been reported in a sample of Mexicans aged 60 and older, where people that brushed their teeth less than twice a day had a higher tooth loss [16]. Our findings confirm that poor oral hygiene can lead to tooth loss [4]. Overall, institutionalized older adults have poor oral hygiene [4, 5]. In Mexico, a study of 139 persons ≥ 60 years old found that only 39.3% of residents in a public nursing home reported brushing their teeth at least once a day [34].

The accumulation of dental biofilm, because of poor oral hygiene, increases the possibility of developing caries and periodontal disease, oral diseases that contribute to tooth loss [4, 5, 10]. Daily tooth brushing with fluoride toothpaste is effective for dental biofilm control [35]. With the use of toothbrush and dental floss, it is possible to remove dental biofilm mechanically, while with toothpaste and mouthwash, it is possible to perform chemical control of dental biofilm [34–37].

Therefore, the most important need for older adults residing in nursing homes is to perform oral hygiene every day [4, 5, 35]. A study carried out in Taiwan among adults aged over 65 demonstrated that having good oral hygiene practices, including regular tooth brushing, was associated with having more remaining teeth [38]. Oral hygiene practices are essential to prevent oral diseases, keep teeth in the mouth, and maintain good oral health [4, 34, 36, 37]. Besides, it is necessary to continuously train personnel to perform tasks related to oral care and caries prevention with the use of silver diamine fluoride [35].

A limitation of the present study that must be taken into account for the correct interpretation of the results is its cross-sectional design, since we can only discuss about associations and not a causal effect (temporal ambiguity). Another limitation observed is related to the type of sampling, which may not be representative of the entire population of that age in the communities where the home nursing centers are located. Subsequent studies should consider the impact of oral diseases, specifically tooth loss, on the quality of life of older adults residing in nursing homes. It is important to encourage the participation of caregivers in the oral hygiene of institutionalized older adults, especially in frail and functional dependent older adults. It is necessary to implement oral health education and promotion strategies, with the intention of improving oral health status and, therefore, the quality of life of the older adults residing in nursing homes.

## 5. Conclusions

In conclusion, we can mention that the tooth loss experience in this sample of older adults was high, on average more than 50% of permanent dentition. Demographic (age) and habit of behavior (tobacco use and less frequent tooth brushing) were associated with an increased tooth loss experience.

## Figures and Tables

**Table 1:** Distribution of the number of lost teeth by demographic, socioeconomic, and behavioral characteristics in a sample of institutionalized older adults in Mexico, 2019.

Variables	Frequency	(%)	Number of lost teeth mean ± sd (median)	*p* value
Gender				
Male	101	(39.3)	17.77 ± 9.68 (19)	
Female	156	(60.7)	19.43 ± 8.59 (22)	0.273^†^
Marital status				
Without partner	229	(89.5)	19.06 ± 8.93 (21)	
With partner	27	(10.5)	16.37 ± 10.01 (14)	0.187^†^
Schooling				
Higher than secondary	63	(24.5)	16.68 ± 9.46 (15)	
Secondary and less	194	(75.5)	19.46 ± 8.83 (22)	0.034^†^
Gerontological center				
AM	100	(38.9)	19.89 ± 8.86 (23)	
VGT	88	(34.2)	18.65 ± 9.51 (20.5)	
LT	22	(8.6)	18.18 ± 7.78 (18)	
OT	47	(18.3)	16.93 ± 9.05 (17)	0.191^‡^
Health insurance				
No	176	(69.3)	20.15 ± 8.69 (22)	
Yes	78	(30.7)	18.08 ± 9.20 (19.5)	0.078^†^
Current medical treatment				
No	34	(13.5)	19.44 ± 9.10 (22.5)	
Yes	217	(86.5)	18.45 ± 9.07 (20)	0.614^†^
Smoking				
No	230	(89.8)	18.26 ± 9.18 (20)	
Yes	26	(10.2)	23.26 ± 6.54 (28)	0.007^†^
Former smoker				
No	137	(53.9)	17.89 ± 9.31 (19)	
Yes	117	(46.1)	19.80 ± 8.69 (22)	0.093^†^
Snacking between meals				
No	95	(37.1)	19.36 ± 8.84 (21)	
Yes	161	(62.9)	18.41 ± 9.20 (20)	0.477^†^
Frequency of tooth brushing				
≥2 times a day	117	(45.9)	13.53 ± 7.02 (13)	
<2 times a day	138	(54.1)	23.24 ± 8.21 (28)	<0.001^†^
Dental care in the last 12 months				
No	66	(26.2)	20 ± 8.38 (22)	
Yes	186	(73.8)	18.35 ± 9.33 (21)	0.247^†^
Severe health problems that prevent hygiene/care				
No	225	(90.7)	18.99 ± 9.10 (21)	
Yes	23	(9.3)	18.08 ± 8.88 (19)	0.585^†^

Note: AM = Arturo Mundet; VGT = Vicente García Torres; LT = Los Tamayo; OT = Olga Tamayo. ^†^Mann–Whitney, ^‡^Kruskall–Wallis.

**Table 2:** Crude and adjusted negative binomial regression models for the number of tooth loss in Mexico, 2019.

	Crude model			Adjusted model		
	Coefficient	% change (IRR)	*p* value	Coefficient	% change (IRR)	*p* value
*Demographic variables*						
Age (years)	0.0151	1.52 (0.46–2.59)	0.005	0.0083	0.83 (0.04–1.64)	0.039
Sex						
Male	1^∗^			1^∗^		
Female	0.0894	9.36 (-4.82–25.35)	0.199	0.1775	19.43 (-4.97–49.73)	0.124
*Socioeconomic variables*						
Schooling						
More than secondary	1^∗^			1^∗^		
Secondary and less	0.1541	16.67 (8.38–25.58)	<0.001	0.1012	10.65 (0.27–22.11)	0.044
*Behavioral variables*						
Smoking						
No	1^∗^			1^∗^		
Yes	0.2423	27.42 (17.71–37.93)	<0.001	0.2452	27.78 (8.17–50.96)	0.004
Frequency of tooth brushing						
≥2 times a day	1^∗^			1^∗^		
<2 times a day	0.5406	71.70 (60.99–83.12)	<0.001	0.5015	65.13 (53.47–77.68)	<0.001

^∗^Reference category. Note: the model was adjusted for the variables that appear in the table. Confidence intervals were calculated with standard errors considering the clusters (AM, VGT, LT, and OT). Interactions were tested but none obtained a value of *p* < 0.15; for this reason, they were not included in the model.

## Data Availability

The datasets generated during and/or analyzed during the current study are available from the corresponding authors on reasonable request.
